# Mild catalytic defects of tert rs61748181 polymorphism affect the clinical presentation of chronic obstructive pulmonary disease

**DOI:** 10.1038/s41598-021-83686-z

**Published:** 2021-02-22

**Authors:** Jialin Xu, Diego Madureira de Oliveira, Matthew A. Trudeau, Yang Yang, Jessica J. Y. Chin, Don D. Sin, Andrew J. Sandford, Judy M. Y. Wong

**Affiliations:** 1 Faculty of Pharmaceutical Sciences, University of British Columbia, 2405 Wesbrook Mall, Vancouver, BC V6T 1Z3 Canada; 2 Universidade de Brasília, Brasília, DF Brasil; 3 Division of Epidemiology and Biostatistics, School of Public Health, Hong Kong University, Pokfulam, Hong Kong; 4 Centre for Heart Lung Innovation, University of British Columbia and St Paul’s Hospital, Vancouver, BC Canada

**Keywords:** Chronic obstructive pulmonary disease, Risk factors, Telomeres

## Abstract

Chronic obstructive pulmonary disease (COPD) is a disorder of accelerated lung aging. Multiple pieces of evidence support that the aging biomarker short telomeres, which can be caused by mutations in telomerase reverse transcriptase (*TERT*), contribute to COPD pathogenesis. We hypothesized that short telomere risk-associated single nucleotide polymorphisms (SNPs) in *TERT*, while not able to drive COPD development, nonetheless modify the disease presentation. We set out to test the SNP carrying status in a longitudinal study of smokers with COPD and found that rapid decline of FEV1 in lung function was associated with the minor allele of rs61748181 (adjusted odds ratio 2.49, *p* = 0.038). Biochemical evaluation of ex vivo engineered human cell models revealed that primary cells expressing the minor allele of rs61748181 had suboptimal telomere length maintenance due to reduced telomerase catalytic activity, despite having comparable cell growth kinetics as WT-TERT expressing cells. This ex vivo observation translated clinically in that shorter telomeres were found in minor allele carriers in a sub-population of COPD patients with non-declining lung function, over the 5-year period of the longitudinal study. Collectively, our data suggest that functional *TERT* SNPs with mild catalytic defects are nonetheless implicated in the clinical presentation of COPD.

## Introduction

Telomere biology disorders consist of a spectrum of tissue degenerative disorders that have variable clinical manifestations and severities, but share the same disease etiology—short telomeres^1^. Telomeres are DNA–protein structures located at the ends of eukaryotic chromosomes^2^. Telomeres shorten with cell divisions and they limit the proliferation of human cells by inducing cellular senescence when a critical length is reached. Telomerase is a ribonucleoprotein complex that can copy the template within its RNA component, telomerase RNA (TER), to telomeric DNA by using its own polymerase, telomerase reverse transcriptase (TERT). De novo synthesis of telomeric repeats at chromosome ends by telomerase delays the depletion of telomeres and extends the proliferation lifespan^3^.

At the cellular level, mean telomere length is dynamically determined by the inherited starting telomere length, the rate of cell turnover, and the rate of telomere synthesis if telomerase is expressed in the cell. Individuals with the shortest telomeres (first percentile) in the general population are susceptible to a spectrum of premature aging disorders^1^, including idiopathic pulmonary fibrosis (IPF), even if they did not carry mutations in known telomere biology genes^4^. At the organismal level, telomere biology disorders, mostly caused by genetic deficiencies in telomerase function, affect tissue regenerative potential and present clinically as diverse disorders. Bone marrow is one tissue with a high proliferative demand that is particularly sensitive to short telomeres, as hematopoietic renewal failure occurs when the replicative potential and the stem cell pool of an affected individual is depleted. Consequently, bone marrow failure diseases, including dyskeratosis congenita and aplastic anemia, are frequently observed in patients with severe telomerase dysfunction.

On the other hand, the lung is a tissue with lower replicative demand but is also frequently affected by telomere dysfunction^1,5^. Patients with mutations in *TERT* suffer from IPF in an autosomal dominant pattern due to telomerase haploinsufficiency and the consequent short telomere-induced tissue renewal defects^6^. Furthermore, IPF is found in up to 20% of all patients with dyskeratosis congenita, the prototype and the first identified telomere biology disorder^7^. Disease presentation in the lung is driven by stem cell proliferative failure following telomere dysfunction in type 2 alveolar epithelial cells (AEC2)^8^.

Chronic obstructive pulmonary disease (COPD) is a multi-factorial trait where inflammation of the peripheral airways and/or loss of elastic recoil due to the destruction of alveoli (emphysema) cause irreversible progressive airflow limitation^9^. Patients with COPD are known to have short telomere length in their peripheral blood leukocytes^10–12^. Notably, the discovery of rare telomerase mutations in emphysema patients was previously reported^13,14^. Stanley et al*.* sequenced the telomerase genes (*TERT* and *TER*) in COPD patients across two large cohorts (COPDgene and the Lung Health Study (LHS)) and discovered that 1% of patients with early-onset severe emphysema had deleterious *TERT* mutations^15^. The prevalence of *TERT* driver mutations in COPD is comparable to alpha-1 antitrypsin deficiency—the only well-characterized genetic cause of emphysema^15,16^. Based on the findings of telomere dysfunction in COPD/emphysema, it has been proposed that stem cells in the lung (i.e., AEC2) were exhausted following cellular senescence during the pathogenesis and progression of COPD^9^.

Single nucleotide polymorphisms (SNPs) in the *TERT* region have been linked to telomere biology disorders, including, but not limited to, IPF^17^ and bone marrow failure^18,19^. Notably, a few non-coding (intronic) SNPs in the *TERT* gene were recently reported to associate with increased risks of COPD in Chinese and Japanese populations^20,21^. While rare, disease-causing *TERT* mutations precipitate the development of tissue regenerative deficiency such as emphysema^22^, in this study, we were interested in understanding whether common *TERT* SNP associated with mild telomere maintenance defects could modify the risk and clinical presentation of COPD.

The non-synonymous *TERT* SNP rs61748181 features a C > T nucleotide substitution, resulting in an amino acid change from alanine to threonine (TERT p.A279T). It is located at a linker domain of the N-terminus of TERT. Targeted deep sequencing has linked this SNP to bone marrow failure^18^, lung cancer^23^ and esophageal cancer^24^. Compared to other disease-associated *TERT* SNPs, it has a relatively high minor allele frequency (MAF) of 3.58%^25^ (Supplementary Table 1). Here, using a combination of human cell models, genetic analysis, and clinical data mining of the LHS cohort, we set out to investigate the effects of *TERT* SNP rs61748181 in the progression of COPD.

**Table 1:** The demographic profile of the LHS population*.

Parameters	rs61748181 CC (N = 2899)	rs61748181 minor allele carriers (CT and TT) (N = 197)
**N (%)**		
Gender (% male)	1957 (67.51)	125 (63.45)
Continuous smokers	1587 (54.74)	119 (60.41)
Intermittent quitters^#^	861 (29.70)	45 (22.84)
Sustained quitters^	451 (15.56)	33 (16.75)
**Mean (SD)**		
Age (years)	48.43 (6.71)	48.49 (6.69)
Pack years	40.10 (17.97)	41.95 (18.69)
FEV1^†^ at baseline	78.26 (9.09)	78.94 (9.00)
FEV1^†^ after 5 years	74.90 (12.35)	75.32 (12.65)

*Caucasian only.

^Sustained quitters are defined as abstinence from all smoked tobacco products at all 5 years of the LHS annual follow up visits (Y1-Y5), confirmed by salivary cotinine and expired carbon monoxide data.

^#^Intermittent quitters consist of self-report smoking history in any annual visits (Y1-Y5); and participants who failed any validation tests. Participants who did not attend a given annual visit were counted as smokers in that visit, and an intermittent quitter.

^†^% of predicted value.

## Results

### rs61748181 carrying status does not affect the overall clinical progression of patients in the LHS cohort

The MAF of rs61748181 in the Caucasians from the LHS population was 3.29% (Table 1). The locus showed a slight deviation from Hardy–Weinberg equilibrium (*p* = 0.040), with a higher number of homozygotes for the minor allele than expected. The deviation is in agreement with a recent report on the excess of homozygotes for rs61748181 minor T allele in COPD patients than in control subjects^26^. 500 out of 3596 samples (13.90%) from Caucasian subjects in the LHS cohort failed the genotyping assay, likely due to DNA sample degradation, as we noted that there were unremarkable differences in baseline characteristics of the demographic profile between the typed and undetermined participants (Supplementary Table 2).

**Table 2:** Characteristics of the study participants in the rapid and non-decliner groups.

Parameters	Rapid decliners (N = 232)	Non-decliners (N = 232)	*p* value*
**N(%)**			
Gender (% male)	136 (58.62)	154 (66.38)	0.1029
**Mean (SD)**			
Age (years)	49.58 (6.53)	47.45 (6.81)	0.0006
Pack years	42.80 (18.67)	38.43 (17.83)	< 0.0001
FEV1 (% predicted) at baseline	74.95 (9.53)	79.72 (7.84)	< 0.0001
FEV1 (% predicted) after 5 years	58.68 (13.20)	82.77 (9.30)	< 0.0001
ΔFEV1 (% predicted/year)	− 3.59 (1.41)	0.45 (1.02)	< 0.0001

*Rapid vs Non-decliners (Fisher’s exact test and two-tailed *t* test).

Statistical analyses were done using the JMP Statistics software package version 13 (SAS Institute Inc.) and R version 3.5.1.

We sorted the Lung Health Study participants by annualized change (i.e., the slope) of FEV1 over 5 years (ΔFEV1_5yrs_) and tested the association between rs61748181 and disease progression by a multivariate linear regression model. While age and smoking (both smoking history in pack-years and smoking status during the 5 years of the LHS study) affected disease progression, we did not observe any significant difference in ΔFEV1_5yrs_ between the rs61748181 minor allele carriers (CT and TT) (n = 197) and those with the CC genotype (n = 2899) (Supplementary Table 3). The lack of correlation between rs61748181 carrying status and ΔFEV1_5yrs_ suggested that COPD disease progression is not likely to be primarily driven by the carrier status of rs61748181.

**Table 3:** The odds ratios of *TERT* SNPs rs61748181 among rapid versus non-decliners in the LHS.

	rs61748181 minor allele carriers	WT	rs61748181 minor allele frequency (%)	Crude OR (95% CI)	Adjusted OR (95% CI)^#^	*p* Value^$^
Rapid decliners	18	214	7.76	2.70 (1.11–6.60)	2.49 (1.01–6.14)	0.038
Non-decliners	7	225	3.02	Referent	Referent	

^#^The odds ratio adjusted for age and smoking history.

^$^*p* value fast versus non-decliners (CC versus CT + TT) for adjusted OR.

Statistical analyses were done using the JMP Statistics software package version 13 (SAS Institute Inc.) and R version 3.5.1.

### Higher prevalence of rs61748181 carriers among rapid decliners of lung function than non-decliners in the LHS cohort

The lack of direct clinical correlation with telomerase genetic defects is not unexpected in telomere biology diseases, as physiological function and tissue renewal are protected by residual telomere length until a critical threshold for telomere dysfunction (unprotection) is reached. We reasoned that when carriers of *TERT* SNPs are subjected to an increased demand for telomere replenishment to maintain tissue integrity, the possible mild defects in telomere maintenance would be exacerbated. Over time, carriers of *TERT* SNPs should experience accelerated deterioration of existing pathological conditions, as a function of the sum total of all etiologic factors that contribute to the loss of tissue integrity, leading to accelerated progression of COPD pathologies^9,27^.

Based on this model, we explored the effect of this *TERT* SNP in patients with extremely rapid disease progression or no progression (Table 2). In a subset of 464 individuals (15% of the LHS population), carriers of rs61748181 TT or CT genotypes were more prevalent among rapid decliners (7.76%) than non-decliners (3.02%), with an adjusted OR of 2.49 (Table 3). In agreement with our model, restricting the comparator populations to patients with extremely rapid disease progression and no progression allowed us to tease out the accumulating biological effect of this *TERT* SNP.

### rs61748181 in *TERT* can lead to compromised telomere length maintenance secondary to reduced telomerase activity

Next, we applied an ex vivo human cell model to assess the telomere maintenance capacity of the minor alleles of rs61748181 and two other *TERT* variants^28^. In addition to rs61748181, we include the telomere biology disorder-associated *TERT* SNP rs377639087, which features the deletion of codon 441 (TERT p.∆E441) and serves as a known telomerase activity defective control. rs112614087 (TERT p.A615T), which has a heterozygosity of 0.500^29^ and therefore is deemed unlikely to change TERT function, was chosen as the variant with normal telomerase activity control. The details of the *TERT* SNPs involved in the biochemical study are summarized in Supplementary Table 1.

**Figure 1:**
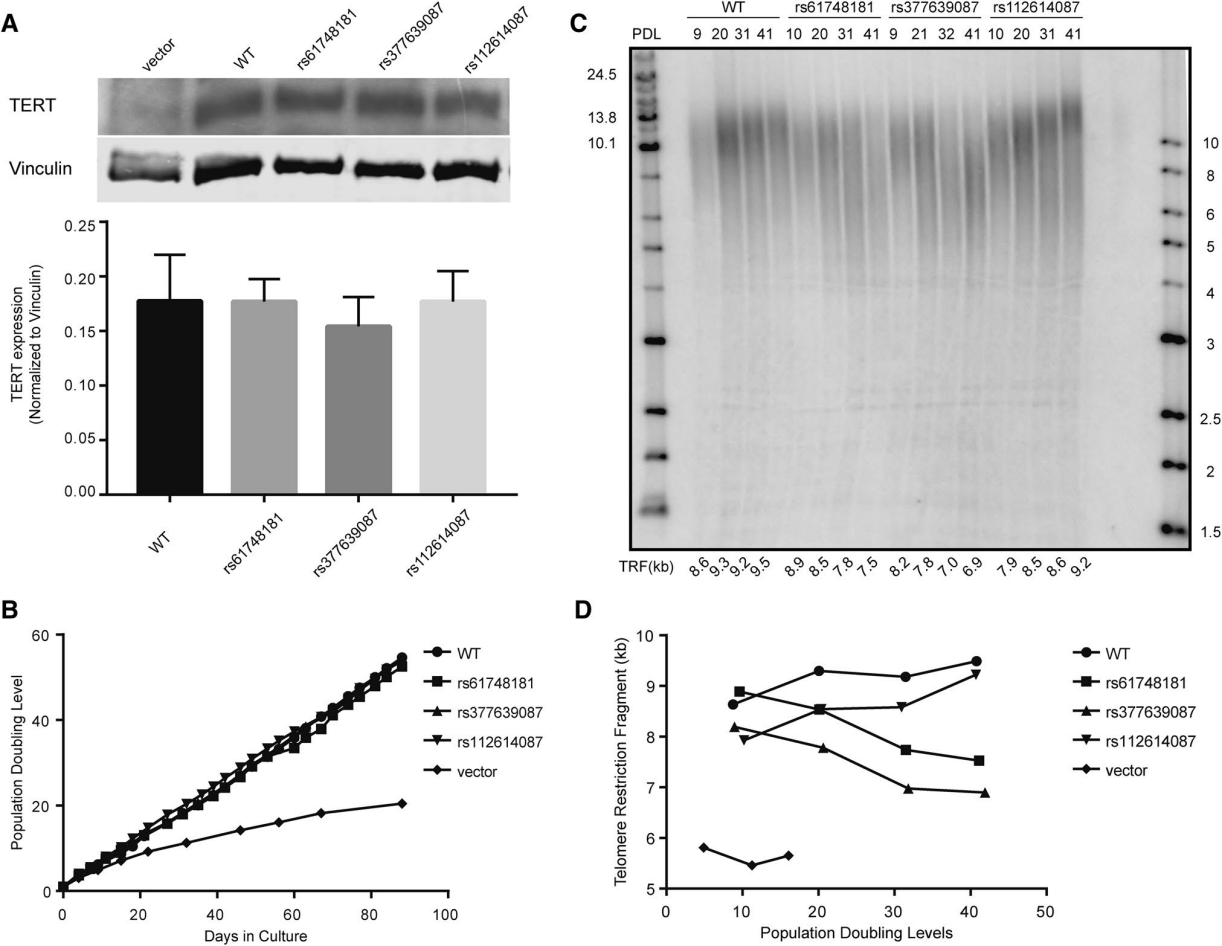
Restoration of recombinant telomerase mediated by the TERT variants in BJ cell lines. (**A**) Recombinant expression of TERT in BJ fibroblasts. TERT expression levels (127 kDa) were normalized to those of vinculin (124 kDa) and the data are presented as mean ± SEM (n = 6). fluorescent & ECL ones). TERT and vinculin signals were differentiated with the use of differently labeled antibodies (chemiluminescent and fluorescent, respectively) and processed accordingly with different detection modalities. Full sets of data were available in the supplementary information (Supplementary Fig. 3) (**B**) Cellular growth kinetics of BJ cell lines expressing recombinant SNP-version of telomerase. (**C**) Telomere lengths in the cells at approximately every 10 PDLs were measured by TRF. Population doubling level (PDL) and telomere lengths (telomere restriction fragments, TRF) for each sample are illustrated. (**D**) Changes in telomere length over cell divisions are plotted.

We used site-directed mutagenesis to introduce the sequences encoded by the minor alleles of the SNPs into the TERT open reading frame (Supplementary Table 4). Like other human fibroblasts, the human foreskin fibroblast BJ cell line silenced its endogenous TERT expression, whereas its TER expression is intact. Stable genomic integration of the coding sequences of TERT (WT and the SNP variants) through infection with retrovirus vectors (pBabe-Hygro-flag3B-TERT) in BJ cells restored telomerase activity. An examination of protein expression revealed that TERT protein levels in these reconstituted cell lines were comparable to each other (Fig. 1A). The population growth kinetics of the TERT-expressing BJ cell lines followed typical logarithmic curves (Fig. 1B). While the BJ vector control cell line (BJ-vector), which does not express telomerase activity, entered replicative senescence following 10 PDLs post-antibiotic selection, all BJ fibroblasts expressing recombinant telomerase (WT/SNP variants) were proliferating continuously in our ex vivo cell model system. Using this ex vivo system, we found the minor allele of SNP rs61748181, when translated into a variant version of TERT (expressed as TERT variant hereafter), was deficient in telomere length maintenance (Fig. 1C,D) while its TERT protein expressions were comparable (Fig. 1A) and growth kinetics remained the same as WT-TERT up to 50–60 PDLs (Fig. 1B).

To understand the mechanism behind defective telomere length maintenance by TERT variants, we applied the primer extension assay to check single nucleotide incorporation (nucleotide addition processivity, NAP) and repeat synthesis (repeat addition processivity, RAP) in WT-TERT and TERT SNP variant cell lines. The RAP activity of the rs61748181-TERT was 94.4 ± 5.5% compared to the WT-TERT (n = 6, *p* = 0.3313) and its NAP activity was 81.1 ± 7.3% of WT (n = 6, *p* = 0.0323) (Supplementary Fig. 1). The RAP and NAP activity of the telomerase activity defective control rs377639087-TERT was 99.41 ± 6.48% (*p* = 0.929) and 71.16 ± 11.22% (*p* = 0.022), respectively. We concluded that the TERT protein encoded by rs61748181 had similar repeat addition capacity as WT-TERT, but reduced nucleotide addition processivity. This minor difference in the polymerase’s processivity translated to the accumulated defects in telomere repeat synthesis overtime, as observed in the cell culture experiments.

### Differences in median telomere length between CC homozygotes and rs61748181 minor allele carriers are evident in subgroups of LHS patients with extreme telomere synthesis demands

**Figure 2:**
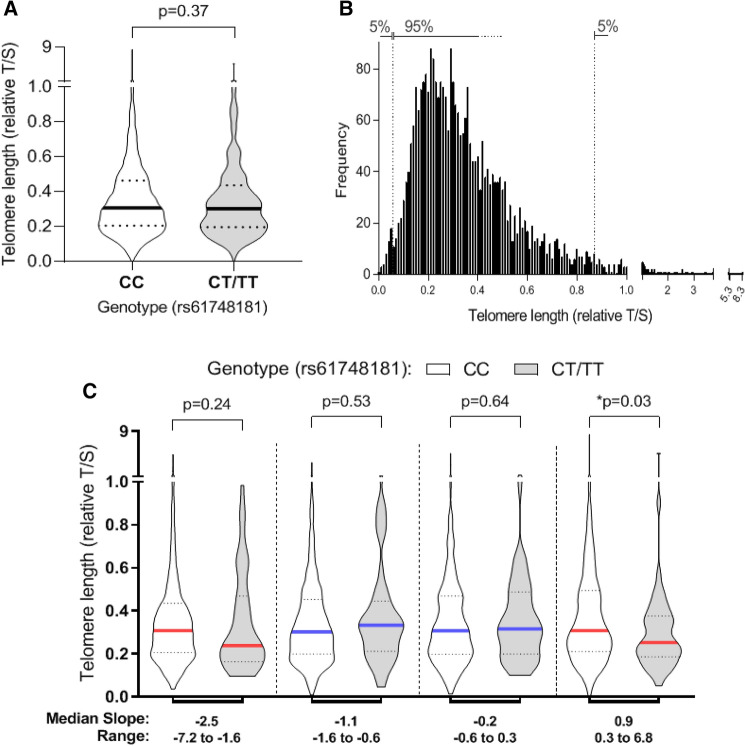
Leukocyte telomere lengths for WT (CC) and rs61748181 carriers (CT/TT) (N = 3050). (**A**) Median telomere length for both WT and rs61748181 carriers showing no significant difference (Mann Whitney test). (**B**) Frequency distribution of relative telomere length among Caucasian members of the LHS cohort (median: 0.306, 95% CI 0.299–0.314). (**C**) Median telomere length of participants sorted based on rs61748181 status (Mann Whitney test). The cohort was divided into quartiles according to disease progression (ΔFEV1_5yrs_); the first quartile represents participants with fastest disease progression, and the fourth quartile indicates those with non-declining lung function. Statistical analyses were performed using the JMP Statistics software package version 13 (SAS Institute Inc.) and R version 3.5.1.

Considering the telomere maintenance defects observed in fibroblasts expressing the minor T allele of rs61748181 over the long term in our cell model, patients carrying the minor allele of rs61748181 should be at risk of increased telomere loss regardless of their COPD progression. We reasoned that we should be able to observe telomere length differences between CC homozygotes and rs61748181 minor allele carriers (genotypes CT and TT) in the LHS population. To test this, we investigated the association between rs61748181 minor allele carrying status and telomere lengths in 3050 genotyped Caucasian patients from the LHS with measurable telomeres. Our analyses did not reveal any statistical differences in median telomere length between rs61748181 minor allele carriers and CC homozygotes (Fig. 2A). Notably, the LHS cohort showed non-normal distribution of telomere length with several participants harboring leukocytes telomere measurements up to forty times longer than the median (Fig. 2B); perhaps not surprisingly, as it is well-known that telomere length regulation is multi-factorial, based on genetic, lifestyle and environmental influences.

We reasoned that the minor telomerase catalysis defects conferred by this SNP would be most evident in the subpopulation of patients with the highest demand for optimal telomere synthesis. Next, we accessed the leukocyte telomere length after dividing the cohort into four groups based on changes in their lung function over 5 years (ΔFEV1_5yrs_). In each group, the leukocyte telomere length for the rs61748181 minor allele carriers was compared to their CC counterparts. In the fourth quartile (Fig. 2C), consisting of patients with no observable decline in their lung function over 5 years monitoring period (positive ΔFEV1_5yrs_), carriers of rs6178181 minor allele (n = 43) had shorter leukocyte telomere lengths than CC patients (n = 719). This difference in telomere lengths based on SNP carrying status was statistically significant (*p* = 0.03). Similarly, rs6178181 minor allele carriers (n = 45) in the first quartile (where patients had the fastest disease progression and most negative ΔFEV1_5yr_ values) tended to have shorter leukocyte telomere lengths than CC patients (n = 717), though statistical significance was not reached. The difference in telomere length between rs6178181 minor allele carriers and CC individuals diminished in the middle subgroups (Fig. 2C), which may explain the lack of correlation between rs61748181 carrier status with telomere length, when the entire LHS cohort was considered (Fig. 2A).

## Discussion

We investigated the biological and clinical effects of a *TERT* SNP rs61748181 which has previously been linked to telomere biology disorders^18^. With clinical samples collected in the LHS, we revealed that carrier status of the SNP rs61748181 aggravated existing disease conditions in COPD patients. Using a combination of molecular and cell biology tools in the laboratory, we further demonstrated that the non-synonymous SNP rs61748181 could lead to defective telomerase catalytic activity and deficient telomere length maintenance. Regression analysis showed that the association was independent of known risk factors such as aging and smoking history. These results implicated *TERT* as a biologically plausible candidate gene for lung function decline and COPD pathogenesis. Not only did our results agree with the previous observation that deleterious mutations in *TERT* contribute to COPD pathogenesis^15^, we also extended this observation to demonstrate that common variants in *TERT* with low penetrance may nonetheless modify disease presentations.

Our ex vivo results demonstrated in a laboratory setting that, when other confounding factors for telomere attrition were fixed, fibroblasts with recombinant expression of the disease-associating TERT variants were predetermined to suboptimal telomere maintenance. However, deficient telomere length maintenance in this context did not translate to a loss of proliferative capacity, as BJ cells expressing the minor allele of rs61748181 were kept in culture for up to 50–60 PDLs, the limit of fibroblast divisions during a lifetime^30^, and demonstrated the same growth rate as WT-TERT expressing cells. To our knowledge, this is the first study to investigate the long-term effects of common TERT variants on telomeres in primary cell lines. Our results confirmed that the short telomere risk-associated SNP rs61748181 encoded a TERT variant that was deficient in telomere length maintenance. We provided strong experimental evidence to support the previous clinical observations that in subjects with bone marrow failure, carriers (heterozygous and homozygous) of this SNP had shorter telomeres than their age-matched peers^18^. These biochemical results are also supported by reports in different cellular and experimental systems^24,31^.

This is the first study both to show the increased risk of rapid lung function decline in COPD patients carrying rs61748181 minor allele and to investigate the association of this SNP with short leukocyte telomeres in patient subsets from in a large cohort study of COPD. The current model of cellular senescence-based COPD pathogenicity^9^ stipulates that the carrier’s stem cell compartments would remain proliferative until their telomeres were too short and trigger stem cell exhaustion, which finally presented as rapid decline and poor lung performance at the end stage of the study. Telomeres shorten at similar rates in blood and somatic tissues^32^. Despite variation, telomere lengths correlate among most human tissue types, including lung tissues^33,34^. Notably, it is recently reported that carriers with rare variants in *TERT,* though not showing telomere biology disorders, have shorter telomere length across all tissues^34^. Therefore, our measurement of leukocyte telomere lengths in COPD patients should be representative of the lengths in their lung tissues.

We also considered the potential influence from a collider effect in our observed correlation between the rs61748181 minor allele carrier status with short leukocyte telomere length. For the collider effect to be true, two variables (telomere length and carrier status of rs61748181) are independently associated with an observation (COPD disease progression, in this case), but they should not be associated. Given that we have biological and empirical reasons to believe short telomere lengths are predicted by the rs61748181 minor allele, and we did not see an association between telomere length and COPD disease progression, we concluded that the collider effect is not relevant here. Finally, by stratifying our analyses to disease progression subgroups, the collider effect interference, if there is one, should be minimized.

An important limitation of our study is a lack of a validation cohort to confirm the clinical effects of rs61748181 carrying status. To reliably determine lung disease progression, longitudinal follow-up and repeated observations are necessary. The impediments from these resource-intensive follow-up procedures are compounded by the low allelic frequencies of rs61748181, requiring a large sample size to enroll in the study. While we do not have access to clinical samples from such a clinical study in lung function decline, we contend that the effects of rs61748181 should apply to other chronic tissue degenerative disorders. Our data should provide the impetus to include *TERT* non-synonymous SNPs in disease progression assessments.

**Figure 3:**
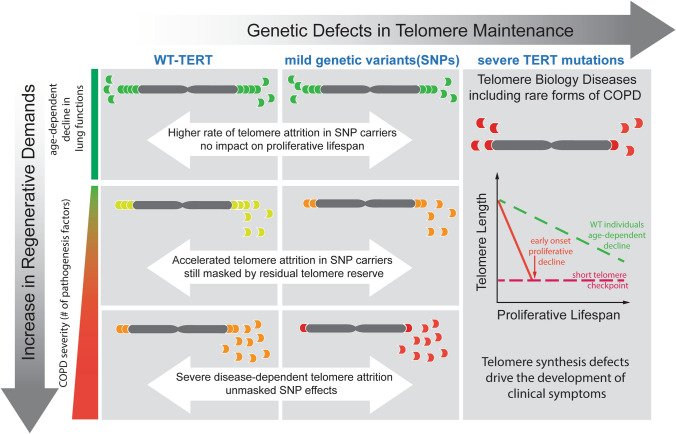
The impact of genetic defects in telomere maintenance on lung function deterioration. Telomere Biology Diseases (TBD, right panel) are caused by rare genetic mutations that severely compromise the synthesis and/or the structural protection of the telomeres. TBD patients display extremely short telomeres at a young age, precipitating the onset of clinical symptoms. In contrast, carriers of common TERT genetic variants (SNPs) (middle panel) are not expected to display pathogenesis associated with short telomeres in the absence of additional factors (environmental, pathological or in combination with other genetic determinants) that accelerate telomere attritions. Even so, these carriers are still genetically predetermined to a decreased capacity to maintain their telomeres, when compared with noncarriers (left panel). These deficiencies, while silent phenotypically within the general population, could be unmasked by the massive increase in regenerative demand associated with tissue degenerative conditions, as seen in the case of COPD. Our work provides a proof-of-principle for novel epidemiological and clinical evaluations of the common SNPs in telomere biology genes on their effects as genetic modifiers rather than drivers of chronic tissue-degenerative disorders.

An individual’s telomere length is determined by multiple factors, including genetics, inherited telomere length, lifestyle and environmental factors^35^. While the effect of environmental insults, such as smoking or infection, was highly variable and subject to huge inter-individual and temporal variations, the effects represented by the inherited telomere length and from the genotype of telomere-related genes are fixed. The comprehension of these variable and fixed effects could facilitate understanding of the pathogenesis and progression in complex disorders. Carriers with genetic variants of low penetrance in telomere biology genes could remain asymptomatic, while the telomere length maintenance defects keep accumulating as long as telomere length does not reach the threshold level. When these individuals encountered other fixed and/or variable factors that accelerate cellular turnover and telomere loss, such as exposure to cell death stimulants including smoking or recurrent infections, the defects in telomerase catalysis would be exacerbated. Over time, carriers of *TERT* SNPs should experience accelerated worsening of existing pathological conditions, as a function of the sum total of all etiologic factors that contributed to the loss of tissue integrity, leading to earlier onset or progression of multiple aging-related diseases, such as cancer^27^, IPF^9^, or COPD^9^ (Fig. 3).

In contrast with our ex vivo model of rs61748181, where we observed telomere maintenance defects along the entire time course of the study, shorter telomeres were only observed in carriers of rs61748181 with COPD progression in the fourth quartile (non-decline in lung function group). We contend that while telomerase catalysis defects and accelerated telomere shortening should be consistent in carriers of rs61748181 within the LHS population, each individual’s variable factors (pathological factors and disease progression rate) could mask this direct correlation. Accordingly, we did not observe any cohort-wide association between rs61748181 with shorter telomere length. The moderate differences in telomere catalysis between CC homozygotes and rs6178181 minor allele carriers in a population could only be unmasked when the effects of these variable factors are removed, or approaching a fixed rate. We reasoned that maximum telomere attrition can be evident in the patient subgroup with the fastest disease progression profile. When we examined the subgroup of patients with the most negative ΔFEV1_5yrs_, rapid lung tissue loss due to the end stage of the pathological processes indeed translated to a trend of shorter telomeres in carriers of rs61748181 minor allele than in CC patients, although statistical significance was not reached. Paradoxically, we demonstrated statistically significant difference in median telomere lengths between carriers of rs61748181 minor allele and CC-individuals, in a subgroup of patients with the most positive ΔFEV1_5yrs_. Higher prevalence of smoking cessation (Supplementary Fig. 2) in this sub-group contributed to the stabilization of lung functions and reduced the lung tissue renewal demand that were associated with rapid telomere attrition. As well, reduction of direct oxidative damage on telomeres from cigarette smoke may also mitigate a significant environment variable in telomere length maintenance. We contend that the reduction in both (pathological and environmental) variables in telomere length regulation provided the opportunity to unmask the catalytic defects of rs61748181 minor allele at a population level.

Variants associated with mild penetrance in telomerase deficiency did not lead to cellular senescence in the absence of other confounding factors, and will not, on their own, drive the onset of chronic degenerative disorders. However, in the presence of accelerated cellular turnover, resulting from exposure to chronic environmental irritants and/or other genetic perturbations that affect tissue-specific functions, the presence of these genetic variants in the telomere biology pathway would be combined, resulting in faster telomere attrition. The effect of mildly deficient genetic polymorphisms in telomere biology genes can be seen as modifiers in accelerated COPD disease progression, when the pathogenetic process leads to excessive demand in lung tissue regeneration. Although the understanding of genetic profile in telomere biology genes does not unequivocally predict one’s disease susceptibility, we contend that a thorough understanding of all the determinants that affect cellular regenerative capacity is important in the prognosis and management of all existing chronic tissue degenerative disorders.

## Methods

### COPD patients

This study used clinical data and biological materials from the LHS. The LHS cohort initially recruited 5887 smokers, aged 35–60, with mild airway obstruction across 10 clinical centers in North America and collected their written informed consents^36^. Spirometry was performed annually over a period of 5 years for all study participants and in a subset after 11 years of study initiation as previously described^37,38^. Lung function was assessed as forced expiratory volume in one second (FEV1) % of predicted, i.e., FEV1 adjusted for age, height and sex^39^. We focused our data analyses on self-identified Caucasian patients in the LHS collection (n = 3596), as they constituted the major ethnicity group in the patient collection, and to avoid the confounding effects from haplotype differences in our analyses. In our study, FEV1 measurements over the 5-year follow-up period (reported as the slope from linear regression of FEV1 values plotted against time, ΔFEV1_5yrs_) was used as a readout for changes in lung function and a measurement of COPD progression. For subgroup analyses based on disease progression, we restricted our study to 15% of the Caucasian subjects with confirmed genotype data, and included equal numbers of rapid- and slow decliners from each end of the linear regression of the rate of decline. This cutoff was used to capture extreme phenotypes that would be likely to be influenced by genetic factors, as we have done in previous studies. Using this cut-off, the rapid decliner group is restricted to LHS participants with ΔFEV1 lower than − 3.5, the characteristics of these selected subjects are shown in Table 2. All experiments were reviewed and performed in accordance to an Institutional Review Board-approved cohort study at the University of British Columbia (Providence Health Care Research Ethics Board certificate number H09-02042, ClinicalTrials.gov Identifier: NCT00000568).

### Genotyping

A single polymorphism (rs61748181) from *TERT* was genotyped by TaqMan SNP Genotyping Assay (Applied Biosystems, Carlsbad, CA, USA). Template-free controls and known genotype controls were included in each experiment. In addition, for the purpose of assay quality control, 5% of the samples were typed again to check the repeatability of the genotyping assay. Genotypes were 100% reproducible in these samples. Herein, we refer WT as homozygous genotype for the reference allele (CC), while rs6178181 minor allele carriers are either homozygous or heterozygous carriers of the minor allele of rs61748181 (CT or TT).

### Statistical analysis

Genotype distributions were assessed for Hardy–Weinberg equilibrium. Pearson’s χ^2^ test and student’s t-test or the Mann–Whitney test were used to assess the differences in dichotomous and continuous parameters between patients with different disease progression patterns, as well as participants with and without the minor allele of rs61748181. The frequencies of the alleles and genotypes between groups were analyzed by Pearson’s χ^2^ analysis for 2 × 2 contingency tables for crude odds ratios (ORs) and by multiple logistic regression for adjusted ORs. The ORs and their 95% confidence intervals (CI) were calculated. Multiple logistic regression for ΔFEV1_5yrs_ was performed to test the association with the *TERT* SNP. Confounding factors included age, gender, pack-years of smoking and smoking status. Statistical analyses were done using the JMP Statistics software package version 13 (SAS Institute Inc.) and R version 3.5.1. Figures were generated by GraphPad Prism 7.0 version (La Jolla, CA, US).

### Cell culture

The primary human foreskin fibroblast BJ cell line was obtained from ATCC. All genetically-modified primary cell lines were cultured in DMEM high-glucose medium (Gibco, ThermoFisher Scientific, Waltham, MA, USA) with 15% fetal bovine serum (Gibco) and were maintained at 37 °C with 5% CO_2_. Population doubling levels (PDLs) were calculated based on the following equation: PDL = (log Xe − log Xb)/0.3 + S, where Xe was the cell harvest number, Xb was the inoculum number, and S was the starting PDL.

### Retroviral gene delivery

The retroviral protein expression vector pBabe-hygro-Flag3B-WT-hTERT (a gift from Dr. Kathleen Collins) was used as the template for *Dpn*I-mediated site-directed mutagenesis. Primers for the mutagenesis were synthesized by IDT DNA (Coralville, IA, USA) and are listed in Supplementary Table 4. The fidelity of positive clones was confirmed with Sanger sequencing of the entire TERT coding sequence (Genewiz, South Plainfield, NJ, USA). Silent mutations were introduced in each pair of primers to create a restriction site for easy identification of positive clones through restriction digestion with a selected endonuclease. The restriction enzymes of choice are also listed in Supplementary Table 4. Retroviral infection was performed by the 293 T cell-based system^40^ to integrate TERT (both WT and variant) coding sequences into the BJ genome. Hygromycin selection at 50 μg/ml was applied to select for positive clones for at least 14 days. Positive clones were pooled for continuous culture. Biological repeats of cell lines creation and long-term measurements for telomere maintenance dynamics were performed.

### Telomere length measurement

The Southern blot-based terminal restriction fragment analysis (TRF) was applied to determine average telomere lengths in cell populations^41^. Data from TRF blots were analyzed by ImageQuant Software (GE Healthcare, Piscataway, NJ, USA) using weighted average analyses^41^. Data were reported as the nearest kilobase using the 1 kb DNA ladder (New England Biolabs, Beverly, MA, USA) as a reference.

Telomeres in peripheral blood leukocytes in the LHS cohort were measured by qPCR as previously described^11^.

### Protein extraction

Cells were removed from culture flasks with trypsin/EDTA. Cell numbers in 100 μL of suspension were counted by Coulter Counter (Beckman Coulter, Brea, CA, USA) according to the manufacturer’s instruction and total cell numbers were calculated accordingly. Following the 2 × wash with ice-cold PBS, cells were resuspended in hypotonic lysis buffer (20 mM HEPES pH 8.0, 2 mM MgCl_2_, 0.2 mM EGTA, 10% glycerol, 1 mM DTT, and 0.1 mM PMSF). Cell suspensions were subjected to four consecutive freeze–thaw cycles from liquid nitrogen to a 37 °C water bath. NaCl was then added to the lysate in two parts to a final concentration of 400 mM. Cell suspensions were incubated on ice for 15 min before clearing the lysate by centrifugation at 13,200 rpm for 15 min. The supernatant (whole cell lysate, WCL) was transferred to a fresh tube, aliquoted, and stored in − 80 °C for further analysis. Protein concentrations were measured by Bradford protein assay.

### Telomerase activity

Telomerase activity was measured by the classical radioactive primer extension assay^42^ using protein extracts from primary BJ cell lines expressing recombinant TERT isoforms. The quantifications of telomerase catalysis and repeat addition processivity were previously described^43^.

### Immunoblot

100 μg WCL were resolved in 5% SDS-PAGE gel. A standard western blotting protocol was applied for the detection of TERT expression in the cell lysates. A sheep anti-human TERT polyclonal antibody (0.5 μg/ml, Abbexa, Cambridge, UK) and a Donkey anti-sheep horseradish peroxidase-conjugated antibody (0.2 μg/ml, Jackson ImmunoResearch, West Grove, PA, USA) were sequentially applied before detection by ECL (PerkinElmer, Waltham, MA, USA) on Kodak X-ray films. The cytoskeletal protein vinculin was used as a normalization control. It has a molecular weight of 124 kDa, which is similar to TERT (127 kDa). Rabbit anti-human vinculin monoclonal antibody (0.1 μg/ml, Cell Signaling, Danvers, MA, USA) and Alexa Fluor 680 Dye (ThermoFisher Scientific, Waltham, MA, USA) were applied before detection by Licor Odyssey CLx Infrared Imaging System (LI-COR Biotechnology, Lincoln, NE, USA) and quantification by ImageJ software (NIH, Bethesda, MD, USA).

## Research ethics approval

This study utilized patient materials collected from an Institutional Review Board-approved cohort study at the University of British Columbia, with informed consent. All experiments in the study were reviewed and performed in accordance to the guidelines provided by the same Institutional Review Board at the University of British Columbia (Providence Health Care Research Ethics Board certificate number H09-02042, ClinicalTrials.gov Identifier: NCT00000568).

## Supplementary Information

Supplementary Information 1.
