# Supplementary material for: Mild catalytic defects of tert rs61748181 polymorphism affect the clinical presentation of chronic obstructive pulmonary disease
Source: Sci Rep. 2021 Feb 22;11:4333. doi: 10.1038/s41598-021-83686-z (PMC7900122; doi:10.1038/s41598-021-83686-z)
Supplement: Supplementary file 1 — Supplementary Information 1. [file 41598_2021_83686_MOESM1_ESM.pdf]

MILD CATALYTIC DEFECTS of TERT rs61748181 POLYMORPHISM AFFECT THE CLINICAL PRESENTATION OF CHRONIC OBSTRUCTIVE PULMONARY DISEASE

Jialin Xu[1], Diego Madureira de Oliveira[1,2], Matthew A. Trudeau[1], Yang Yang[3], Jessica J.Y. Chin[4], Don D. Sin[4], Andrew J. Sandford[4], Judy M.Y. Wong[1]

[1] Faculty of Pharmaceutical Sciences, University of British Columbia, Vancouver, BC, Canada

[2] Universidade de Brasília – Brasília (DF), Brasil

[3] Division of Epidemiology and Biostatistics, School of Public Health, Hong Kong University, Hong Kong.

[4] Centre for Heart Lung Innovation, University of British Columbia and St Paul's Hospital, Vancouver, BC, Canada

Corresponding author:

Dr. Judy Wong

judy.wong@ubc.ca

Faculty of Pharmaceutical Sciences, University of British Columbia

2405 Wesbrook Mall, Vancouver BC, V6T 2G9, Canada

Supplementary Table 1. ***TERT*** **SNPs involved in the study**

AMINO ACID CHANGE	REFSEQ	NUCLEOTIDE CHANGE	DBSNP NUMBER	OVERALL MINOR Allele	OVERALL MAJOR Allele	CAUCASIANS MINOR Allele	CAUCASIANS MAJOR Allele	MAF Overall	MAF Caucasians	FUNCTIONAL DOMAIN	DATA SOURCE
**A279T**	NM_198253.2	835G>A	rs61748181	48	4960	36	970	0.958%	3.579%	between TEN and TRBD	1000 Genomes Project Phase 3
**ΔE441**	NM_198253.2	1323_1325 delGGA	rs377639087	4	5004	4	1002	0.080%	0.398%	TRBD	1000 Genomes Project Phase 3
**A615T**	NM_198253.2	1843G>A	rs112614087	Not Available						RT	NCBI dbSNP database

Supplementary Table 2. Demographic profile of the entire LHS cohort, with comparison between genotyped and undetermined samples.

	ENTIRE COHORT		GENOTYPED		UNDETERMINED		
	**N**		**N**		**N**		
**NUMBER OF SUBJECTS**	3741		3225	86.21%	516	13.79%	
**AGE (RANGE, MIN - MAX)**	34	67	34	62	35	67	
**GENDER (% MALE)**	2367	63.27%	2026	62.82%	341	66.09%	
**RACE (% CAUCASIAN)**	3596	96.12%	3096	96.00%	500	96.90%	
**CONTINUOUS SMOKERS (%)**	2067	55.25%	1787	55.41%	280	54.26%	
**INTERMITTENT QUITTERS (%)**	1075	28.74%	935	28.99%	140	27.13%	
**SUSTAINED QUITTERS (%)**	598	15.99%	502	15.57%	96	18.60%	
	**Mean**	**SD**	**Mean**	**SD**	**Mean**	**SD**	**p value**
**AGE (YEARS)**	48.53	6.69	48.44	6.71	49.23	6.23	0.013
**PACK YEARS**	40.5	18.3	40.2	18.0	41.8	19.9	0.016
**FEV1 (% OF PREDICTED VALUE) AT BASELINE**	78.32	9.05	78.25	9.07	78.75	8.91	0.23
**FEV1 (% OF PREDICTED VALUE) AFTER 5 YEARS**	74.97	12.34	74.83	12.36	75.91	12.19	0.060
**ΔFEV1 (% OF PREDICTED VALUE) OVER 5 YEARS**	-0.99	1.89	-1.00	1.86	-0.90	1.77	0.25

Supplementary Table 3. Distribution of changes in FEV1 over 5 years ($\Delta FEV1_{5yrs}$) in rs61748181 minor allele carriers and WT individuals.

	WT (CC)	rs61748181 minor allele carriers (CT and TT)
10% PERCENTILE	-3.271	-3.022
25% PERCENTILE	-1.97	-1.973
MEDIAN	-0.9	-0.8364
75% PERCENTILE	0.12	0.0925
90% PERCENTILE	1.02	1.056
MEAN	-0.9999	-0.9557
STD. DEVIATION	1.981	2.774
STD. ERROR OF MEAN	0.03698	0.1982
LOWER 95% CI OF MEAN	-1.072	-1.347
UPPER 95% CI OF MEAN	-0.9274	-0.5649

Supplementary Table 4. Primer design for site-directed mutagenesis.

Primer #	*dbSNP ID*		*Sequence (5' – 3')*
TERT A279T with EagI-F	rs61748181	Forward	GTG GTG TCA CCT GCC CGG CCG ACC GAA GAA GCC ACC TCT
TERT A279T with EagI-R		Reverse	AGA GGT GGC TTC TTC GGT CGG CCG GGC AGG TGA CAC CAC
TERT E441del with BbsI-F	rs377639087	Forward	GGC GGC CCC CGA GGA AGA CAC AGA CCC CCG T
TERT E441del with BbsI-R		Reverse	ACG GGG GTC TGT GTC TTC CTC GGG GGC CGC C
TERT A615T-HincII-F	rs112614087	Forward	GAA GCC AGG CCC ACC CTG TTG ACG TCC AGA C
TERT A615T-HincII-R		Reverse	GTC TGG ACG TCA ACA GGG TGG GCC TGG CTT C

Supplementary Figure 1. Non-synonymous SNP variants in *TERT* affect telomerase activity and processivity in a SNP variant-specific manner (A) Expression of variant TERT proteins in BJ cell lines in the primer extension assay (PAGE and autoradiography). (B) The activity of SNP variant versions of telomerase relative to WT-TERT. (C) Processivity calculated by the linear regression method. Data are presented as mean ± SEM (n = 6).

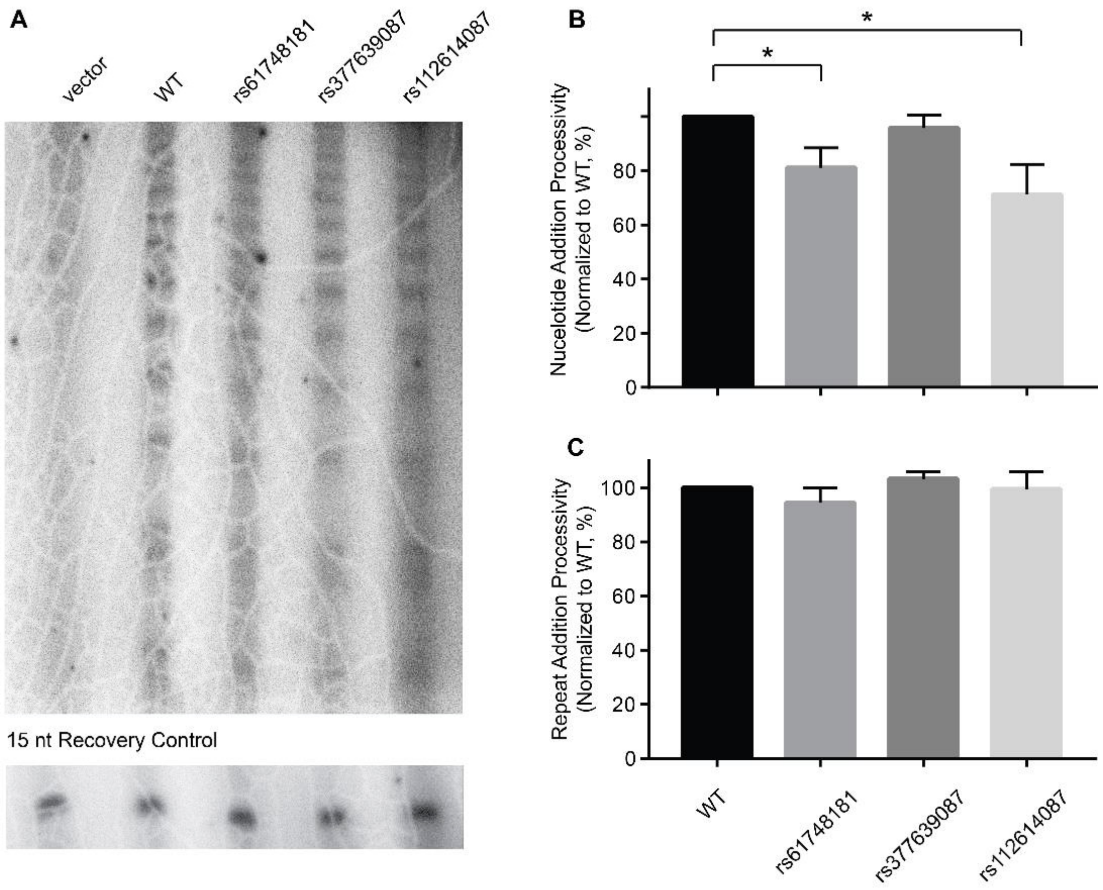

Supplementary Figure 2. Smoking status of LHS participants. Continuous smoker did not quit smoking ever and individuals in the *non-continuous* category include both intermittent and sustained quitters. The LHS cohort was divided into quartiles according to disease progression ($\Delta FEV1_{5yrs}$); the first quartile represents participants with fastest disease progression, and the fourth quartile indicates those with non-declining lung functions. The proportion of continuous smoker are negatively correlated with lung function over 5 years (X^2 test, p<0.0001). Statistical analyses were done using the JMP Statistics software package version 13 (SAS Institute Inc.) and R version 3.5.1.

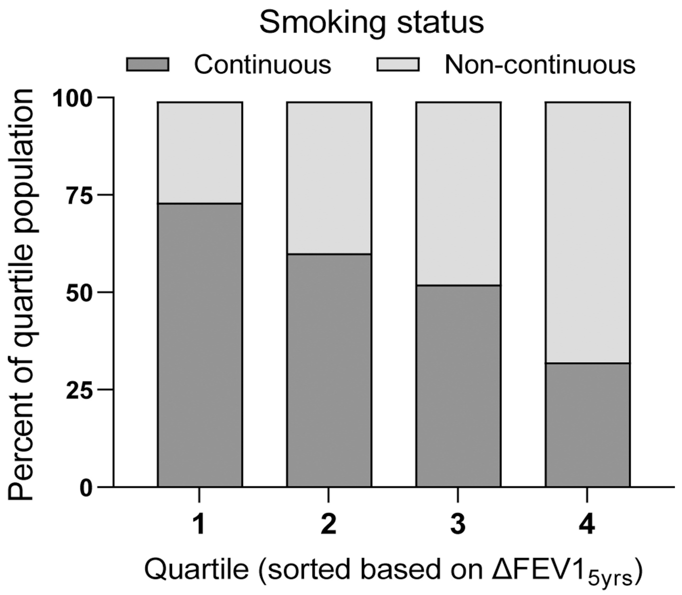

Supplementary Figure 3. Uncropped images of TERT and vinculin protein detection by western blotting in Figure 1.

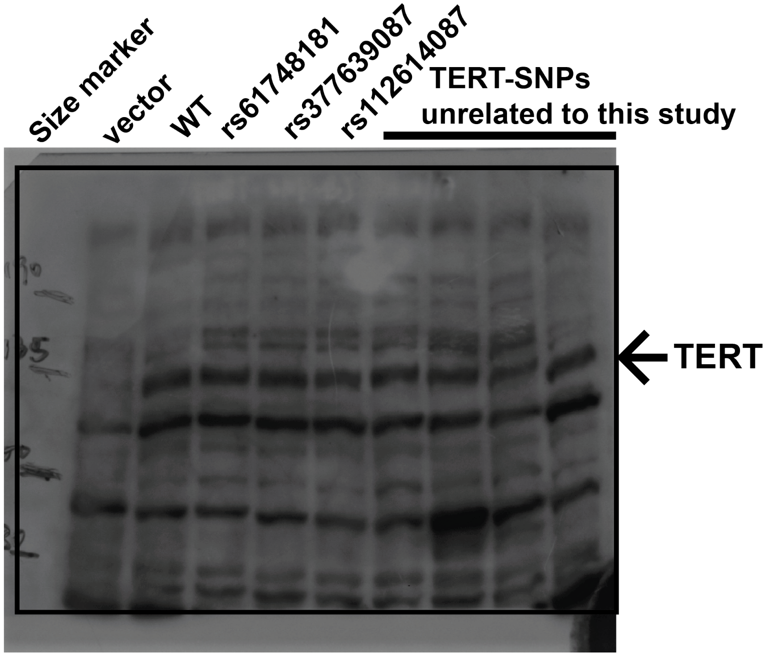

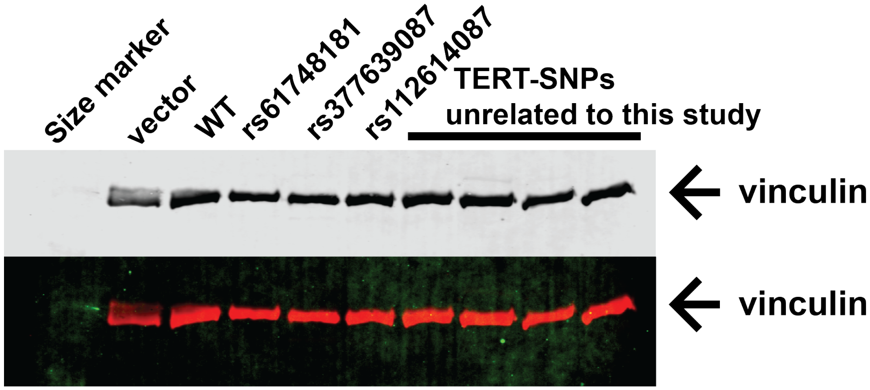

References

1. Swift S, Lorens J, Achacoso P, Nolan GP. Rapid production of retroviruses for efficient gene delivery to mammalian cells using 293T cell-based systems. Curr Protoc Immunol. 2001;Chapter 10:Unit 10.7C.
2. Killedar A, Stutz MD, Sobinoff AP, Tomlinson CG, Bryan TM, Beesley J, et al. A Common Cancer Risk-Associated Allele in the hTERT Locus Encodes a Dominant Negative Inhibitor of Telomerase. PLoS Genet. 2015;11(6):e1005286.
3. Zeng XL, Thumati NR, Fleisig HB, Hukezalie KR, Savage SA, Giri N, et al. The accumulation and not the specific activity of telomerase ribonucleoprotein determines telomere maintenance deficiency in X-linked dyskeratosis congenita. Hum Mol Genet. 2012;21(4):721-9.
4. Lee J, Sandford AJ, Connett JE, Yan J, Mui T, Li Y, et al. The relationship between telomere length and mortality in chronic obstructive pulmonary disease (COPD). PLoS One. 2012;7(4):e35567.
5. Wong JM, Collins K. Telomerase RNA level limits telomere maintenance in X-linked dyskeratosis congenita. Genes Dev. 2006;20(20):2848-58.
6. Hukezalie KR, Thumati NR, Côté HC, Wong JM. In vitro and ex vivo inhibition of human telomerase by anti-HIV nucleoside reverse transcriptase inhibitors (NRTIs) but not by non-NRTIs. PLoS One. 2012;7(11):e47505.
7. Latrick CM, Cech TR. POT1-TPP1 enhances telomerase processivity by slowing primer dissociation and aiding translocation. EMBO J. 2010;29(5):924-33.
